# Traditional knowledge-based lifestyle interventions in the prevention of obesity and type 2 diabetes in Indigenous children in Canada: a systematic review protocol

**DOI:** 10.1186/s13643-019-0961-4

**Published:** 2019-03-06

**Authors:** Rebecca Crawford, E. Danielle Sims, Kuan-Wen Wang, Michael Youssef, Ajantha Nadarajah, Angelica Rivas, Laura Banfield, Lehana Thabane, M. Constantine Samaan

**Affiliations:** 1 Department of Pediatrics, McMaster University, 1280 Main Street West, HSC-3A57, Hamilton, Ontario L8S 4K1 Canada; 2 Division of Pediatric Endocrinology, McMaster Children’s Hospital, Hamilton, Ontario Canada; 3 Indigenous Undergraduate Summer Research Scholars Program, Hamilton, Ontario Canada; 4 Medical Sciences Graduate Program, McMaster University, Hamilton, Ontario Canada; 5 Health Sciences Library, McMaster University, Hamilton, Ontario Canada; 6 Department of Health Research Methods, Evidence and Impact, McMaster University, Hamilton, Ontario Canada; 7 Department of Anesthesia, McMaster University, Hamilton, Ontario Canada; 8 Centre for Evaluation of Medicines, St. Joseph’s Health Care, Hamilton, Ontario Canada; 9 Biostatistics Unit, St. Joseph’s Healthcare, Hamilton, Ontario Canada

**Keywords:** Systematic review, Indigenous, Pediatric, Intervention, Primary prevention, Type 2 diabetes mellitus

## Abstract

**Background:**

Approximately 50% of all youth-onset type 2 diabetes mellitus (T2DM) in Canada occurs in Indigenous children. In adults, cardiovascular disease is one of the leading causes of mortality in First Nations communities, and diabetes is a significant contributor to the risk of developing this disorder. The early onset of diabetes may predispose these children to premature cardiovascular disease and influence their longevity and quality of life. As a result, the implementation of culturally tailored obesity and T2DM primary prevention programs is vital. This systematic review aims to assess the effectiveness of existing traditional knowledge-based lifestyle intervention programs on preventing obesity and T2DM in Indigenous children in Canada.

**Methods:**

We will conduct database searches in MEDLINE, Embase, PsycINFO, SPORTDiscus, CINAHL, Web of Science, Cochrane Database of Systematic Reviews, and the Cochrane Controlled Register of Trials. We will also conduct grey literature searches of central repository of trials (ClinicalTrials.gov), ProQuest Dissertations, Theses A&I, and Indigenous studies portal research tools. Reviewers will independently review titles, abstracts, and full-text articles retrieved from databases to assess potentially eligible studies, and relevant articles will be assessed for risk of bias and quality. The primary outcomes include the change in body mass index *z*-scores or a diagnosis of diabetes. The secondary outcomes include the change in measures of adiposity as well as lifestyle and metabolic profiles. A meta-analysis will be performed if two or more studies have used similar study designs, comparable intervention techniques , similar populations and measured similar outcomes.

**Discussion:**

This review will provide a summary of current interventions to prevent obesity and T2DM in Indigenous children in Canada and help determine the gaps in the literature so that interventions can be developed to control the surge in pediatric T2DM in Indigenous communities.

**Systematic review registration:**

PROSPERO CRD42017072781

**Electronic supplementary material:**

The online version of this article (10.1186/s13643-019-0961-4) contains supplementary material, which is available to authorized users.

## Background

The rates of pediatric obesity and type 2 diabetes mellitus (T2DM) are rising in Indigenous communities in North America [1, 2]. Indigenous children in Canada have one of the highest rates of pediatric T2DM in the world [3, 4, 5] and constitute approximately 50% of new onset T2DM cases with intergenerational effects including antenatal exposure to maternal obesity and diabetes programming some of this risk [2, 4]. The increase in T2DM is mainly driven by obesity [6], which manifests early in life and rises with aging in these communities. As preschoolers, 11% of Indigenous children in North America are overweight, and these rates increase rapidly to 40% in the 9–13 year age group [7, 8]. There are several drivers of diabetes and obesity epidemics in Indigenous communities, including historical and social factors related to the legacy of colonialism, cultural suppression, and systemic poverty [3, 11, 12]. These factors interact with genetic and environmental elements that may further augment the rise in T2DM prevalence over the past few decades [9–12].T2DM contributes to adverse outcomes including premature mortality and comorbidities including nephropathy, neuropathy, and retinopathy along with cardiovascular diseases in Indigenous communities [1]. While it is imperative to prevent or delay the onset of T2DM and its comorbidities, incorporation of traditional knowledge into the design, deployment, and evaluation phases of interventions in communities will likely maximize success and improve buy-in.

Traditional knowledge is a system of information, skills, experiences, and beliefs that is assembled over time and is passed on from one generation to the next, thus becoming part of that community’s identity. Traditional interventions that may have an impact on obesity and diabetes may encompass different components such as dancing, hunting, ceremonies, storytelling, food harvesting and storage, and teachings in an attempt to address the physical, mental, emotional, and spiritual domains of health [13]. This systematic review aims to assess the effectiveness of traditional knowledge-based lifestyle interventions in preventing obesity and T2DM in Indigenous children in Canada.

### Research question

In Indigenous children in Canada, are traditional knowledge-specific lifestyle interventions effective in preventing obesity and T2DM?

### Outcomes

#### Primary

For obesity, we will assess the change in body mass index (BMI) z-score before and after the intervention. The development of diabetes will be measured based on a fasting blood glucose level of ≥ 7.0 mmol/L or a random blood glucose or blood glucose taken 2 h post-oral glucose tolerance test of ≥ 11.1 mmol/L [14].

#### Secondary

We will report on changes in total adiposity (defined as fat mass percentage) and central adiposity defined by measuring waist circumference, waist-to-hip ratio, and waist-to-height ratio. We will also include changes in diet and physical activity, as well as metabolic profile changes including lipids, Hemoglobin A1c, glucose, and insulin levels.

## Methods

The methods used in this protocol paper are reported in accordance with the statement for the Preferred Reporting Items for Systematic Review and Meta-Analysis Protocols (PRISMA-P) [15] (Additional file 1).

### Literature search

We will collaborate with a senior Health Sciences Librarian to develop the search strategy. We will conduct database searches in MEDLINE, Embase, Web of Science, Cochrane Controlled Register of Trials (CENTRAL), PsycINFO, SPORTDiscus, CINAHL, and the Cochrane Database of Systematic Reviews (DSR). A sample search strategy for MEDLINE is reported in Table 1.

**Table 1:** MEDLINE search strategy

No.	Searches
1	Obesity/
2	Pediatric Obesity/
3	obes*.mp.
4	over weight*.mp.
5	overweight*.mp.
6	Overweight/
7	Body Mass Index/
8	BMI.mp.
9	body mass.mp.
10	body fat distribution/
11	adiposity/
12	body weight/
13	waist circumference/
14	waist-height ratio/
15	skinfold thickness/
16	waist-hip ratio/
17	body fat.mp.
18	adipos*.mp.
19	skinfold*.mp.
20	skin fold*.mp.
21	(waist adj3 (ratio* or circumference*)).mp.
22	body weight*.mp.
23	or/1-22
24	exp Diabetes Mellitus, Type 2/
25	type 2 diabet*.mp.
26	T2DM.mp.
27	T2D.mp.
28	NIDDM.mp.
29	non-insulin dependent diabet*.mp.
30	((adult onset or matur* onset) adj3 diabet*).mp.
31	Glucose Intolerance/
32	(glucose adj3 (toleran* or intoleran*)).mp.
33	or/24-32
34	23 or 33
35	adolescent/
36	child/
37	child, preschool/
38	child*.mp.
39	adolescen*.mp.
40	youth*.mp.
41	teen*.mp.
42	preteen*.mp.
43	pre-teen*.mp.
44	preadolescen*.mp.
45	p?ediatric*.mp.
46	or/35-45
47	early medical intervention/
48	intervention*.mp.
49	preventive health services/
50	“early intervention (education)”/
51	Early Medical Intervention/
52	health education/
53	school health services/
54	Diet/
55	Diet, Diabetic/
56	Diet Therapy/
57	diet*.mp.
58	Exercise/
59	exercise therapy/
60	educat*.mp.
61	exercis*.mp.
62	school.mp.
63	Public Health/
64	public health.mp.
65	community based.mp.
66	Health Behavior/
67	Health Promotion/
68	behav*.mp.
69	exp sports/
70	sport* program.mp.
71	sports.mp.
72	(physical* adj2 (fit or fitness or activ*)).mp.
73	la crosse.mp.
74	lacrosse.mp.
75	hockey.mp.
76	run*.mp.
77	basketball*.mp.
78	soccer.mp.
79	baseball.mp.
80	canoe*.mp.
81	kayak*.mp.
82	danc*.mp.
83	activity.mp.
84	active.mp.
85	country food*.mp.
86	traditional food*.mp.
87	exp Life Style/
88	life style*.mp.
89	lifestyle*.mp.
90	Medicine, Traditional/
91	(traditional adj2 (medicine* or knowledge or practice* or game*)).mp.
92	medicine wheel*.mp.
93	Ceremonial Behavior/
94	ceremon*.mp.
95	song or songs or sing or singing).mp.
96	Singing/
97	Religion/
98	prayer*.mp.
99	spirituality/
100	Culture/
101	(culture or cultural).mp.
102	(wellbeing or well being).mp.
103	community participat*.mp.
104	nutrition*.mp.
105	primary prevention/
106	prevent*.mp.
107	Motor Activity/
108	“Play and Playthings”/
109	((play or plaything* or toys or toy) adj3 child*).mp.
110	or/47-109
111	health services, Indigenous/
112	Indians, North American/
113	Inuits/
114	First Nations.mp.
115	(native* adj2 Canad*).mp.
116	inuk*.mp.
117	metis.mp.
118	or/111-117
119	aboriginal*.mp.
120	indigenous.mp.
121	first people*.mp.
122	indian*.mp.
123	or/119-122
124	exp Canada/
125	canad*.mp.
126	alberta*.mp.
127	quebec*.mp.
128	britishcolumbia*.mp.
129	BC.mp.
130	manitoba*.mp.
131	new brunswick*.mp.
132	newfoundland*.mp.
133	labrador*.mp.
134	nova scotia*.mp.
135	nunavut*.mp.
136	ontario*.mp.
137	prince edward island*.mp.
138	PEI.mp.
139	yukon*.mp.
140	northwest territor*.mp.
141	or/124-140
142	123 and 141
143	118 or 142
144	34 and 46 and 110 and 143
145	remove duplicates from 144

A search strategy will also be developed to search grey literature databases including ClinicalTrials.gov and ProQuest Dissertations and Theses A&I. In addition, the interdisciplinary Indigenous studies portal research tool (iPortal), a collection of articles, books, theses, and other documents, focusing primarily on research about Indigenous communities in Canada, will also be included in the search [16]. The searches will be updated closer to completion of the systematic review to ensure all relevant evidence is collected.

All relevant articles identified during full-text screening will have their reference lists searched to determine if there are any cited relevant studies. Endnote X8 [17] will be used to combine all studies retrieved from the databases. We will export these files into an Excel spreadsheet and remove duplicates. The remaining titles, abstracts, and full texts will be screened for relevance. The reviewers will record all decisions regarding eligibility in the spreadsheet.

### Study selection and eligibility criteria

This systematic review will be completed by three teams of two independent reviewers who will be responsible for screening the titles, abstracts, and full texts of the articles. At each stage, reviewers are required to make independent decisions regarding the relevance of the article to the research question and inclusion criteria. The team will come to a consensus regarding the inclusion or exclusion of articles at each stage through discussions. If there are disagreements that were not resolved through discussions, the final decision will be made by a third reviewer. Each team will also perform risk of bias and quality assessments on the abstracted articles.

This systematic review will include studies of boys and girls, less than 18 years of age, and who are of First Nations, Inuit, and Métis descent. Only Indigenous children in Canada will be included, and we will not exclude studies based on language, setting, or timeline of publication. We will exclude children with a current diagnosis of T2DM, as this review aims to assess prevention studies.

In studies that include children of other ethnicities, we will abstract relevant data exclusively related to Indigenous children and will contact the Principal Investigators to obtain the data if they were not reported separately. If there is a control group of Indigenous children who did not receive the intervention, we will include their data as well. For studies involving community-based interventions, we will include data specific to the pediatric sub-population taking part in the study and will contact the Principal Investigators if data for children are not reported separately.

**Fig. 1:**
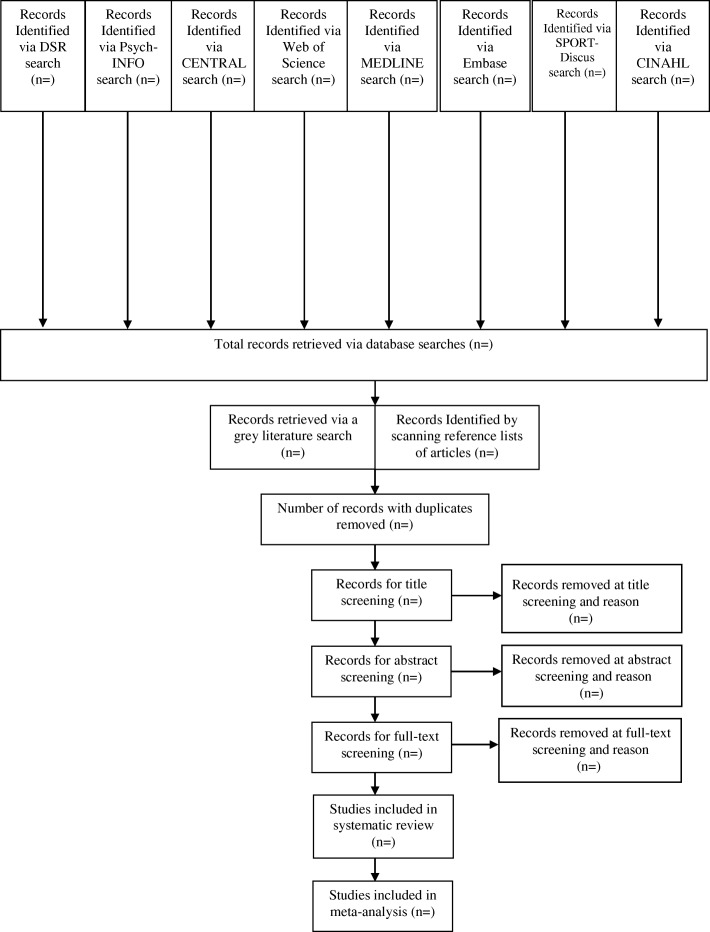
Article screening process flow chart

We will include primary research of randomized controlled trials, non-randomized trials, and uncontrolled before-and-after study designs [18]. Such primary research articles must include the assessment of the effectiveness of traditional knowledge-based lifestyle interventions using the primary and secondary outcomes noted above. A flow diagram will be used to document the screening process [19, 20] (Fig. 1).

### Data collection

The information from the articles will be entered into a data abstraction form specifically created for this systematic review. Different sections will document study details including title, first author, publication year, journal name, setting (academic, hospital, community, other), the University or Institutional affiliation, primary and secondary outcomes, inclusion/exclusion criteria, study design/duration, details of the implemented methods for intervention, and sample size at the start and the end of the intervention. We will also collect data on the age, sex, and community group designation.

We will also include data regarding the Nation/Reserve, province/territory, number of participants in the control group if applicable, description of the home, community and general environment if reported, any experience of discrimination, bullying, trauma or co-morbid psychiatric disorders, baseline BMI z-score and percentile, adiposity measures including fat mass percentage reported by using Dual Energy X-Ray Absorptiometry or bioelectrical impedance. In addition, central adiposity clinical measures will be collected if available including waist circumference, waist-to-hip ratio, and waist-to-height ratio. We will also collect data on lifestyle changes including diet and physical activity profiles, and any alteration in metabolic profiles including blood glucose, plasma insulin, Hemoglobin A1c, and lipid profile including total cholesterol, high-density lipoprotein, low-density lipoprotein, and triglycerides. We will also gather data on family history of obesity and diabetes and the presence of other medical conditions.

### Risk of bias and quality assessment

Several tools will be used to assess the risk of bias depending on the study design. For randomized controlled trials, the Risk of Bias Assessment Tool developed by the Cochrane Collaboration will be used which will report the study as having either a low, high, or unclear risk of bias [21]. Using this tool, studies will be assessed across six domains including sequence generation, allocation concealment, blinding, incomplete data, selective reporting outcomes, and other sources that may increase the potential risk of bias.

For non-randomized studies, the Risk of Bias in Non-Randomized Studies-I (ROBINS-I) tool will be used to assess the risk of bias over three domains [22]. The first domain evaluates risk of bias before the intervention takes place and specifically determines bias introduced by confounding factors and participant selection. For this particular review, confounding factors may include age at which intervention took place, sex, family history of diabetes, adiposity, Nation/Reserve, geographic location, community characteristics, and metabolic comorbidities. The second domain of this tool assesses the risk of bias as the intervention is taking place and evaluates the bias introduced due to a misclassification of intervention type. The final domain determines the risk of bias introduced after the intervention takes place. Biases may include missing data, methods of outcome measurements, selective data reporting, and any discrepancy between the proposed and implemented interventions.

Another tool used to assess the risk of bias of studies involving a before-and-after intervention design is a checklist developed by the University of Alberta Evidence-Based Practice Centre (UAEPC) [23]. This tool evaluates the risk of bias introduced by incomplete data, selection bias, and methods of outcome measurement. All studies will have their risk of bias assessed by two independent reviewers who will discuss and come to a consensus, with a third reviewer to be involved to arbitrate disagreements.

The Grading of Recommendations, Assessment, Development and Evaluation (GRADE) guidelines will be utilized to assess the quality of the reported evidence from the research articles included in the review [24]. This tool determines the quality of evidence based on the risk of bias, inconsistency, indirectness, imprecision and publication bias. This information will be combined into tables using the GRADEpro software and confidence in the ovall evidence base will be reported as high, moderate, low, or very low [25].

### Statistical analysis

A meta-analysis will be completed if two or more studies with similar study design, methods, populations and would have assessed similar outcomes are identified. When dichotomous outcomes occur, they will be reported as an odds ratio. Continuous outcomes will be stated as the difference in means and 95% confidence intervals.

To quantify heterogeneity, we will use the chi squared (χ^2^) test and inconsistency index (*I*^2^) and the Cochrane Collaboration threshold will be used for analysis, with p-value of <0.1 and *I*^2^ of 75% being indicative of heterogeneity [26].

We will conduct a sensitivity analysis if more than ten studies measuring similar outcomes are available. This process will involve the comparison of separately run meta-analyses with one involving the exclusion of studies that may confound the meta-analysis, including those with high risk of bias and small sample size; the other analysis will involve all relevant studies without the exclusion of data. If more than ten studies assessing an outcome are available, publication bias will be evaluated using a funnel plot and Egger’s Test to assess plot asymmetry [27]. Review Manager software version 5.3 [28] will be used for the meta-analysis, in conjunction with Statistical Package for the Social Sciences (SPSS) version 25.0 to conduct Egger’s Test [29].

If a meta-analysis is not possible for the given set of primary research studies, a narrative summary of the data will be reported in addition to data tabulation.

This systematic review will be reported according to guidelines provided by the Preferred Reporting Items for Systematic Reviews and Meta-Analyses (PRISMA) [19, 20], and any amendments to the protocol will be documented and addressed in the review.

## Discussion

With the rising prevalence of obesity and T2DM in Indigenous children in Canada, it is critical that effective interventions are implemented to avert premature adverse cardiometabolic outcomes and diabetes-related complications [1]. Defining and generalizing the implementation of currently available interventions to prevent obesity and T2DM will likely improve outcomes and reduce healthcare costs. If no current interventions are identified to address these two diseases, then the situation requires designing, implementing, and evaluating novel methods to tackle diabetes and obesity in Indigenous communities. If, on the other hand, traditional knowledge-based interventions were found to be successful at preventing obesity and/or diabetes, then urgent investment in the implementation of these interventions is warranted.

One of the strengths of this systematic review is that the team has specific and blended expertise, with a clinician with research methods knowledge (MCS), statistical and methodological expertise (LT), and a Senior Health Sciences Librarian (LB) involved in the design of the search strategy. In addition, the work of student researchers on Indigenous health projects (RC, DS, KWW, MY, AN, AR) will help the development of valuable experience in systematic review methodology and Indigenous health studies.

One of the limitations that may confound the results obtained in this systematic review is the inclusion of Indigenous populations residing only in Canada, although we recognize that colonial-imposed borders do not necessarily limit the mobility of the Indigenous populations across different territories [30]. While this is a potential limitation, the data will have a significant impact on defining the existence of interventions to prevent obesity and T2DM in Indigenous children in Canada, and these results may be generalized to communities across North America.

This systematic review will provide critical insights into the effectiveness of prevention through intervention to limit obesity and T2DM, will help identify gaps in the literature, and pave the way for future research to design implement programs in an attempt to halt these epidemics in Indigenous communities.

## Additional file

Additional file 1: PRISMA-P checklist. This checklist outlines crucial aspects of a protocol paper and designates their location in the manuscript. (DOCX 33 kb)

## Abbreviations

BMI: Body mass index
CENTRAL: Cochrane Controlled Register of Trials
CINAHL: Cumulative Index to Nursing and Allied Health Literature
DSR: Cochrane Database of Systematic Reviews
Embase: Excerpta Medica Database
GRADE: Grading of Recommendations, Assessment, Development and Evaluation
iPortal: The interdisciplinary Indigenous studies portal research tool
MEDLINE: Medical Literature Analysis and Retrieval System
PRISMA: Preferred Reporting Items for Systematic Reviews and Meta-Analyses
PRISMA-P: Preferred Reporting Items for Systematic Review and Meta-Analysis Protocols
ROBINS-I: Risk of Bias in Non-Randomized Studies—of Interventions
SPSS: Statistical Package for the Social Sciences
T2DM: Type 2 diabetes mellitus
UAEPC: University of Alberta Evidence-Based Practice Centre
